# Severity of locomotive syndrome in surgical cervical spondylotic myelopathy patients: a cross-sectional study

**DOI:** 10.20407/fmj.2022-035

**Published:** 2023-05-09

**Authors:** Kurenai Hachiya, Soya Kawabata, Takehiro Michikawa, Sota Nagai, Hiroki Takeda, Daiki Ikeda, Shinjiro Kaneko, Nobuyuki Fujita

**Affiliations:** 1 Department of Orthopaedic Surgery, School of Medicine, Fujita Health University, Toyoake, Aichi, Japan; 2 Department of Environmental and Occupational Health, School of Medicine, Toho University, Ota, Tokyo, Japan; 3 Department of Spine and Spinal Cord Surgery, School of Medicine, Fujita Health University, Toyoake, Aichi, Japan

**Keywords:** Cervical spondylotic myelopathy, Locomotive syndrome, 25-question geriatric locomotive function scale, Japanese orthopaedic association cervical myelopathy evaluation questionnaire

## Abstract

**Objectives:**

Although cervical spondylotic myelopathy (CSM) has a marked impact on locomotive function, few studies have evaluated this relationship in terms of locomotive syndrome. Thus, we assessed (i) the stages of locomotive syndrome in preoperative CSM patients using the 25-question geriatric locomotive function scale (GLFS-25), (ii) the correlation between GLFS-25 scores and the Japanese orthopaedic association (JOA) scores or the JOA cervical myelopathy evaluation questionnaire (JOACMEQ) scores, and (iii) the factors associated with stage 3 locomotive syndrome in preoperative CSM patients.

**Methods:**

We used clinical data from 107 patients scheduled for cervical spinal surgery for CSM. Data were collected prior to surgery, and included age, gender, body mass index, medical history, JOA score, and JOACMEQ and GLFS-25 scores.

**Results:**

Of the included CSM patients, 93.5% were diagnosed with locomotive syndrome, of whom 77.6% were stage 3 according to GLFS-25 evaluation. For the correlation between GLFS-25 and JOA or JOACMEQ, the upper and lower extremity function scores of JOACMEQ and the JOA were strongly inversely correlated with the GLFS-25 score. Finally, multivariate analysis suggested that severe lower extremity status in the JOACMEQ was significantly associated with stage 3 locomotive syndrome in preoperative CSM patients.

**Conclusions:**

Using the GLFS-25, we found that lower extremity status had the strongest association with stage 3 locomotive syndrome in preoperative CSM patients. These findings are useful for preventing CSM patients from requiring future nursing care.

## Introduction

Cervical spondylotic myelopathy (CSM) is a common progressive degenerative disorder that affects the cervical spine, predominantly in middle-aged and older patients.^1^ Age-related degenerative change of the cervical spine is the leading risk factor involved in the pathology of CSM, which includes intervertebral disc bulging, ligamentum flavum hypertrophy, and facet joint degeneration. With the global increase in the aging population,^2^ the incidence of CSM is expected to rise.^3^

A healthy life expectancy represents a long and healthy life to be lived.^4^ The global gap between life expectancy and healthy life expectancy creates a considerable economic and social burden.^4^ To reduce this gap, the Japanese orthopaedic association (JOA) coined the concept “locomotive syndrome.”^5^ Locomotive syndrome refers to impairment of motor functions (e.g., standing up and walking) due to musculoskeletal dysfunction, and it has a high risk of requiring future nursing care.^5^ The stage of locomotive syndrome is assessed on the basis of the stand-up test, two-step test, and the 25-question geriatric locomotive function scale (GLFS-25).^5^ Of these three tests, GLFS-25 is a self-reported comprehensive and simple test requiring no specific space for evaluation. Stages 1 and 2 of locomotive syndrome involve a progressive decline in mobility functions. Recently, stage 3 was added as a condition involving progressive deterioration in mobility function with hindered social participation.^6^

CSM commonly causes gait disturbance due to spinal cord compression, which decreases quality of life and increases the risk of falls.^7^ Additionally, CSM patients can show upper extremity symptoms that commonly involve numbness, pain, sensory disturbance, finger clumsiness, and muscle weakness, which interfere with daily life. Therefore, CSM may have a marked impact on locomotive syndrome. GLFS-25 was recently shown to predict the risk of recurrent falls in postoperative patients with cervical myelopathy.^8^ However, to the best of our knowledge, no studies have evaluated locomotive syndrome in preoperative CSM patients.

Thus, the aims of the present study were to determine the stages of locomotive syndrome in preoperative CSM patients using the GLFS-25, to examine the correlation between GLFS-25 scores and JOA scores or JOA cervical myelopathy evaluation questionnaire (JOACMEQ) scores, and to identify factors associated with stage 3 locomotive syndrome in preoperative CSM patients.

## Methods

### Study participants

This was a cross-sectional study. We collected clinical data from 107 patients scheduled for spinal surgery for CSM at our institution from April 2020 to March 2022. Patients with cervical disc herniation, ossification of the posterior longitudinal ligament, atlantoaxial subluxation, a retro-odontoid pseudotumor, or dropped head syndrome were excluded. Six board-certified spinal surgeons diagnosed CSM using the JOA guidelines.^9^ This study was approved by the Ethics Committee of Fujita Health University (Approval code: HM20-530). Our institution’s ethical review board approved the study and accepted the opt-out consent method, which meant that unless individuals contacted us to withdraw, we included all eligible patients in the study. The study protocol adhered to the tenets of the Declaration of Helsinki.

### Data collection

We collected clinical data prior to surgery, including age, gender, body mass index, medical history, surgical procedure, American society of anesthesiologists physical status, JOA score, and scores for JOACMEQ and GLFS-25. The surgical procedure used for each patient was also examined. The baseline characteristics are shown in Table 1.

### GLFS-25

The GLFS-25 is a self-reported comprehensive measure that assesses impairment in the prior month.^10^ Each item is graded from no impairment (0) to severe impairment (4). Participants answered these questions preoperatively on a paper-based document. The content of the GLFS-25 is shown in Table 2. The stages are determined by the total score: ≥7=stage 1; ≥16=stage 2; and ≥24=stage 3.

### JOACMEQ and the visual analogue scale

The JOACMEQ is comprised of five domains ranging from 0 to 100—these domains include cervical spine function, upper extremity function, lower extremity function, bladder function, and quality of life. The visual analogue scale (VAS) evaluates stiffness in the neck or shoulders, tightness in the chest, numbness in the arms or hands, and numbness from the chest to the toe.

### Statistical analysis

Pearson’s chi-square test was used to assess categorical variables. Pearson’s correlation coefficients were used to evaluate the correlations between the JOACMEQ or VAS and the GLFS-25 score. We calculated the area under the curve based on the receiver operating characteristic curve. We estimated the predicted values using upper extremity and lower extremity function scores from JOACMEQ for stage 3 locomotive syndrome (GLFS-25 score ≥24). The cutoff value was the maximum value of the Youden index (sensitivity+specificity–1). To identify independent risk factors associated with stage 3 locomotive syndrome, we implemented a multivariable Poisson regression model using age, gender, body mass index, American society of anesthesiologists physical status, and the five JOACMEQ domains. We determined the estimated prevalence ratios (PRs) and 95% confidence intervals (95%CI) for stage 3 locomotive syndrome. Poisson regression was performed with statistical software (STATA16; Stata Corporation, College Station, TX, USA). A *P*-value ≤0.05 was considered statistically significant.

## Results

### Evaluation of locomotive syndrome stage of CSM patients using GLFS-25

**Figure 1:**
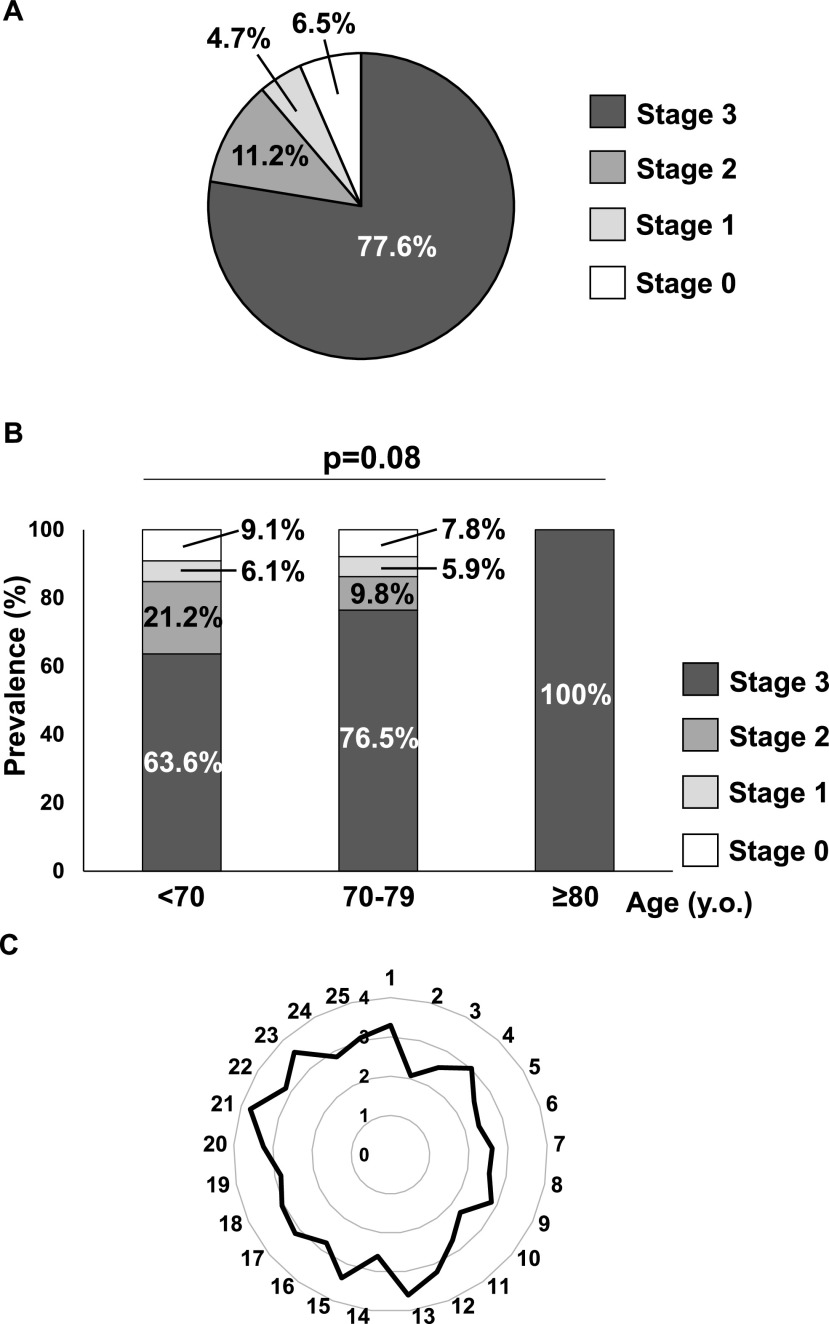
(A) Distribution of stages of locomotive syndrome in all participants assessed using the 25-question geriatric locomotive function scale (GLFS-25). (B) Distribution of stages for locomotive syndrome by age. (C) Average score of each item in GLFS-25.

The distribution of stages of locomotive syndrome in all patients using the GLFS-25 scores is shown in Figure 1A. Of the patients, 93.5% had locomotive syndrome, which involved 77.6% with stage 3, 11.2% with stage 2, and 4.7% with stage 1. When the distribution of locomotive syndrome stages was assessed by age, the prevalence of stage 3 locomotive syndrome increased with increasing age (Figure 1B). The mean score for each GLFS item for all patients is shown in Figure 1C. In the three items (i.e., Q13, Q21, and Q23), the average score was >3.5 points.

### Correlation between GLFS-25 scores and JOA or JOACMEQ scores in CSM patients

**Figure 2:**
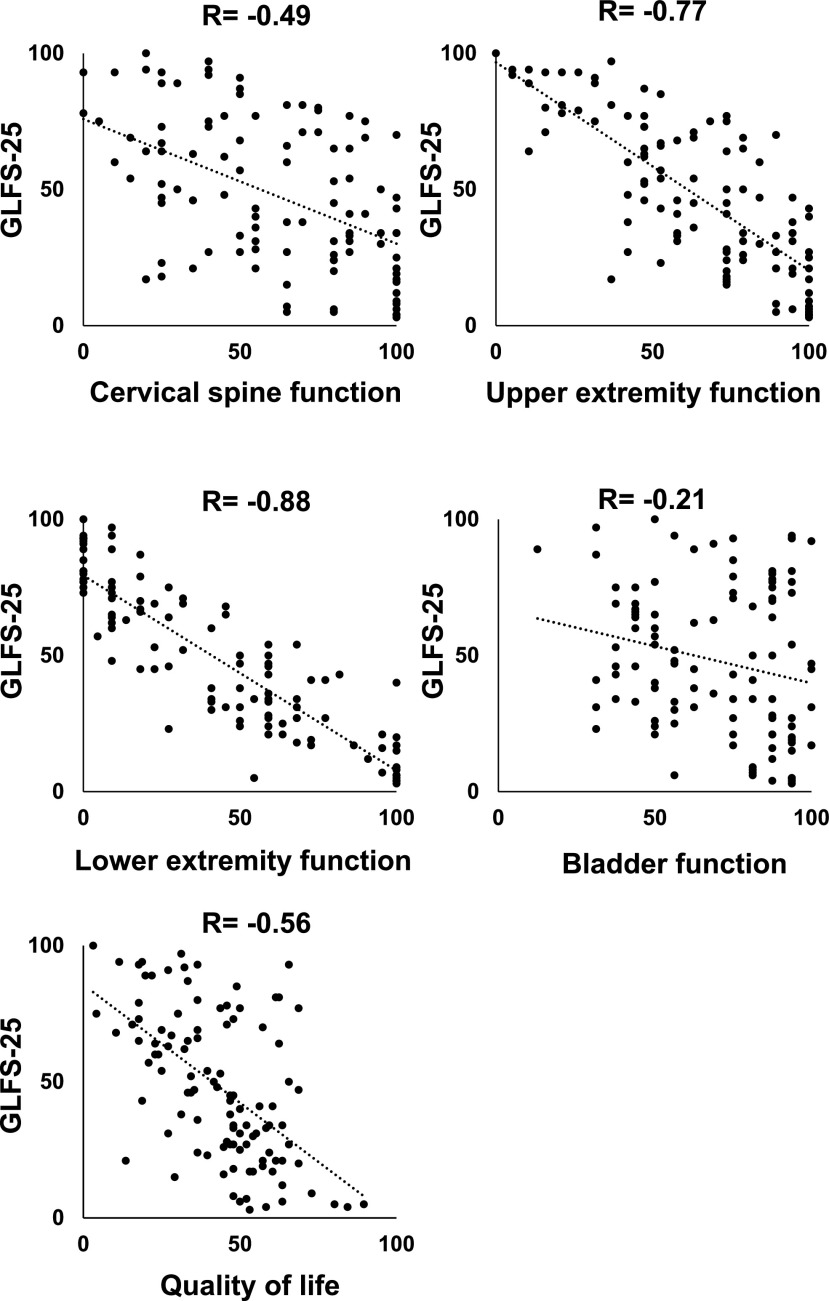
Correlations between the 25-question geriatric locomotive function scale (GLFS-25) and the (A) Japanese orthopaedic association (JOA) score or (B) each domain of the JOA cervical myelopathy evaluation questionnaire (JOACMEQ). Pearson’s coefficient analysis was performed.

**Figure 3:**
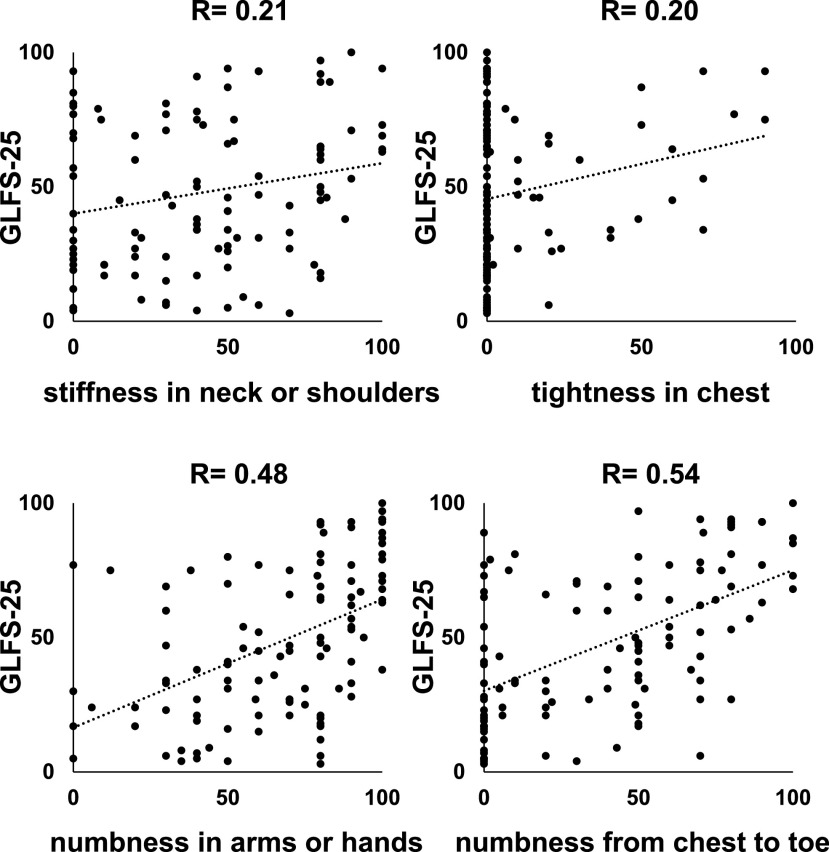
Correlations between the 25-question geriatric locomotive function scale (GLFS-25) and each domain of the visual analogue scale (VAS). Pearson’s coefficient analysis was performed.

We examined the correlation between GLFS-25 scores and JOA, JOACMEQ, or VAS scores. The JOA scores (*r*=−0.80, *P*<0.01) showed a strong inverse correlation with GLFS-25 scores (Figure 2A). The scores of upper extremity function (*r*=−0.77, *P*<0.01) and lower extremity function (*r*=−0.88, *P*<0.01) of JOACMEQ also showed a strong inverse correlation with GLFS-25 scores (Figure 2B). The scores of cervical spine function (*r*=−0.49, *P*<0.01) and quality of life (*r*=−0.56, *P*<0.01) showed a moderate inverse correlation with GLFS-25 scores (Figure 2B). By contrast, there was no correlation between bladder function scores (*r*=−0.21, *P*<0.01) and GLFS scores (Figure 2B). VAS for numbness in the arms or hands (*r*=0.48, *P*<0.01) and numbness from the chest to the toe (*r*=0.54, *P*<0.01) had a moderate positive correlation with GLFS-25 scores (Figure 3). Finally, the VAS for stiffness in the neck or shoulders (*r*=0.21, *P*=0.03) and tightness in the chest (*r*=0.20, *P*=0.03) had no correlation with GLFS-25 scores (Figure 3).

### Identification of the cutoff value of JOACMEQ for stage 3 locomotive syndrome

**Figure 4:**
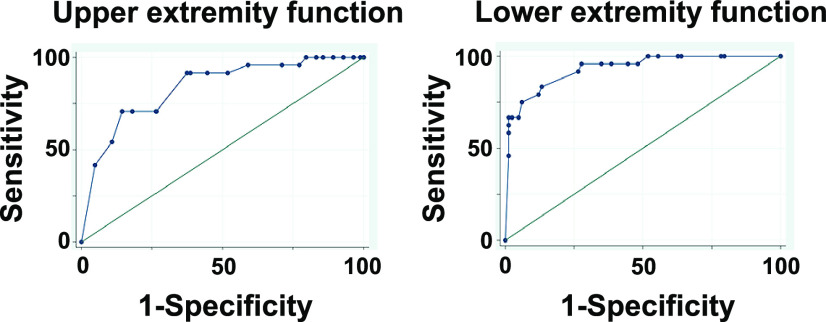
Receiver operating characteristic curve for the cutoff point of upper extremity function and lower extremity function of the Japanese orthopaedic association cervical myelopathy evaluation questionnaire.

We assessed the predictive ability of the sensitivity and specificity of upper and lower extremity function in JOACMEQ for stage 3 locomotive syndrome (Figure 4). The area under the curves of upper and lower extremity function were 0.84 (0.75–0.93) and 0.93 (0.88–0.99), respectively, suggesting that these domains had moderate to strong accuracy (Table 3). The cutoff values for the score of upper and lower extremity function were estimated at 89.5 (sensitivity=70.8%, specificity=85.5%) and 63.6 (sensitivity=83.3%, specificity=86.8%), respectively (Table 3).

### Identification of factors associated with stage 3 locomotive syndrome in CSM patients

Finally, to decrease the influence of confounding factors, multivariable analysis was used to identify factors associated with stage 3 locomotive syndrome in CSM patients. In the multivariate analysis, considering the high prevalence of stage 3 locomotive syndrome between the patients, we used Poisson regression analysis. Statistical analysis showed that lower extremity function (score <14: PR=1.6, 95%CI=1.1–2.4; score >14 to ≤55: PR=1.5, 95%CI=1.1–2.0) was significantly associated with stage 3 locomotive syndrome (Table 4).

## Discussion

The main findings of the present study were that the majority of CSM patients who were eligible for cervical spinal surgery were diagnosed with locomotive syndrome, with approximately 80% showing stage 3 locomotive syndrome according to GLFS-25 evaluation. Compared with a previous study examining locomotive syndrome by age group in the Japanese general population,^11^ we found that the prevalence of locomotive syndrome was obviously high among CSM patients by age group. We also found that the scores of upper and lower extremity function in both the JOACMEQ and the JOA scores had a strong inverse correlation with GLFS-25 scores in preoperative CSM patients. Additionally, statistical analysis determined the cutoff values for detection of stage 3 locomotive syndrome in preoperative CSM patients in the upper and lower extremity function of JOACMEQ. Finally, a severe status of the lower extremity in the JOACMEQ was significantly associated with stage 3 locomotive syndrome in preoperative CSM patients.

Seichi et al. initially developed GLFS-25 as an evaluation tool for early detection of locomotive syndrome,^10^ while several other studies have reported use of GLFS-25 as an assessment tool for lumbar spinal canal stenosis.^12–15^ Araki et al. reported that the GLFS-25 score for lumbar spinal canal stenosis patients was significantly correlated with other commonly reported measures.^13^ Additionally, Kato et al. reported that GLFS-25 was an appropriate tool for assessment of locomotive syndrome in patients with severe musculoskeletal diseases including LSS.^14^ By contrast, there are few reports using the GLFS-25 in CSM, although Kimura et al. found that postoperative CSM patients had an approximately 60% prevalence of locomotive syndrome using GLFS-25, and suggested that GLFS-25 could be used to predict recurrent fall risk.^8^ In the present study, most preoperative CSM patients were diagnosed with locomotive syndrome using GLFS-25. According to our results, the GLFS-25 may also predict a requirement for cervical spinal surgery in CSM patients diagnosed with locomotive syndrome. Because stage 3 locomotive syndrome was defined as a progressive decline in mobility function with hindered social participation, CSM patients with stage 3 locomotive syndrome are at least eligible for cervical surgery.

Although the contents of all 25 items of the GLFS-25 are associated with CSM symptoms, marked changes in the average score of three items (i.e., ‘Difficulty walking briskly,’ ‘Difficulty performing sports activity,’ and ‘Refrain from joining social activities’) were observed in CSM patients in the present study. Thus, these three items should be examined when using GLFS-25 as a screening tool for CSM. Nevertheless, it remains unclear whether these symptoms are improved by cervical spinal surgery. For older patients with degenerative diseases of the lumbar spine and lower extremities, surgery was reported to be beneficial in alleviating locomotive syndrome.^14,16^ Longitudinal studies of CSM patients using GLFS-25 are required to determine whether cervical spine surgery improves locomotive syndrome.

Our multivariable analysis showed that disability of lower extremity function was specifically involved in stage 3 locomotive syndrome in CSM patients. These findings suggest that CSM patients with severe disability of the lower extremity may require future nursing care and interventional treatment. Considering that spastic gait, a clinical feature of CSM, increases the risk of falls and future fractures,^17^ clinicians should be particularly aware of the lower extremity status in CSM patients in terms of healthy life expectancy. Early surgical intervention may be desirable in these patients because the more severe the CSM, the less effective the surgery.^18^

This study has some limitations. First, we did not assess our patients using other locomotive syndrome risk tests such as the stand-up and two-step tests. Thus, we did not have an accurate diagnosis of the stage of locomotive syndrome. Second, this was a single-center study with potential selection bias. Thus, the results of this study should be validated in a multicenter study. Third, our subjects were limited to inpatients with indications for surgery for CSM. Because more patients have conservative therapy than surgery, the present study does not reflect all CSM patients. Finally, although CSM patients commonly have lumbar spondylosis and osteoarthritis of the knee and hip, which can influence the risk level for locomotive syndrome,^12–14,19–21^ the lumbar, knee, and hip regions of our patients were not evaluated using radiography or medical examination. Nevertheless, this study provides useful information for the assessment of locomotive syndrome in CSM patients using the GLFS-25.

In conclusion, we provide new evidence that GLFS-25 can be used in preoperative CSM patients to assess the stage of locomotive syndrome. Most of these patients were diagnosed with stage 3 locomotive syndrome. Additionally, lower extremity status had the highest association with stage 3 locomotive syndrome. This study provides useful information for reducing the requirement for future nursing care in CSM patients.

## Figures and Tables

**Table1:** Baseline characteristics (n=107)

Age (y)	<50	6 (5.6%)
	50–59	15 (14.0%)
	60–69	12 (11.2%)
	70–79	51 (47.7%)
	≥80	23 (21.5%)

Gender	Men	73 (65.5%)
	Women	34 (34.5%)

BMI (kg/m^2^)	<20.0	21 (19.6%)
	20.0–24.9	62 (57.9%)
	≥25.0	24 (22.4%)

Medical history	Hypertension	66 (61.7%)
	Hyperlipidemia	28 (26.2%)
	Diabetes	27 (25.2%)
	Cardiovascular diseases	23 (21.5%)
	Stroke	10 (9.3%)
	Malignancy	11 (10.3%)

Surgical procedure	Posterior decompression surgery	89 (83.2%)
	Posterior decompression and fusion surgery	4 (3.7%)
	Anterior decompression and fusion surgery	12 (11.2%)
	Anterior decompression and fusion surgery+posterior decompression surgery	1 (0.9%)
	Anterior decompression and fusion surgery+posterior decompression and fusion surgery	1 (0.9%)

BMI, body mass index.

**Table2:** Content of the 25-question risk assessment

Q1	Pain in neck or upper limbs
Q2	Pain in back or buttocks
Q3	Pain or numbness in lower limbs
Q4	Painful to move body in daily life
Q5	Difficulty getting up from bed or lying down
Q6	Difficulty standing up from a chair
Q7	Difficulty walking inside the house
Q8	Difficulty putting on and taking off a shirt
Q9	Difficulty putting on and taking off pants
Q10	Difficulty using the toilet
Q11	Difficulty washing the body in the bath
Q12	Difficulty going up and down stairs
Q13	Difficulty walking briskly
Q14	Difficulty keeping yourself neat
Q15	Walking distance without rest
Q16	Difficulty going out to visit neighbors
Q17	Difficulty carrying objects weighing approximately 2 kg
Q18	Difficulty using public transportation
Q19	Difficulty doing simple tasks and housework
Q20	Difficulty doing load-bearing tasks and housework
Q21	Difficulty performing sports activity
Q22	Refrain from meeting friends
Q23	Refrain from joining social activities
Q24	Fall-related anxiety
Q25	Anxiety about being unable to walk in the future

**Table3:** Cutoff value for stage 3 of locomotive syndrome

	AUC	Cutoff value	Sensitivity (%)	Specificity (%)
Upper extremity function	0.84 (95%CI=0.75–0.93)	89.5	70.8	85.5
Lower extremity function	0.93 (95% CI=0.88–0.99)	63.6	83.3	86.8

AUC, area under the curve; 95%CI, 95% confidence interval.

**Table4:** Poisson regression model for stage 3 of locomotive syndrome

			Number of patients	Number of stage 3	Prevalence of stage 3 (%)	*P*-value	Crude model					Multivariate model*			
							PR	95%CI		*P*-value		PR	95%CI		*P*-value
Age	<65		27	15	55.6		Reference					Reference			
	65–74		33	27	81.8		1.5	1.0	2.1	0.04		1.3	0.9	1.7	0.11
	≥75		47	41	87.2	<0.01	1.6	1.1	2.2	0.01		1.2	0.9	1.7	0.15

Sex	Women		34	31	91.2		Reference					Reference			
	Men		73	52	71.2	0.02	0.8	0.7	0.9	<0.01		0.9	0.8	1.1	0.23

BMI (kg/m^2^)	<25.0		83	65	78.3		Reference					Reference			
	≥25.0		24	18	75.0	0.73	1.0	0.7	1.2	0.74		1.0	0.8	1.3	0.79

ASA-PS	1		17	11	64.7		Reference					Reference			
	2 or 3		90	72	80.0	0.17	1.2	0.9	1.8	0.26		0.9	0.6	1.3	0.61

JOACMEQ	Cervical spine function	Tertile 1 (<45)	34	29	85.3		1.3	1.0	1.7	0.04		0.8	0.6	1.1	0.22
		Tertile 2 (45–75)	31	27	87.1		1.4	1.0	1.8	0.02		1.0	0.7	1.2	0.72
		Tertile 3 (>75)	42	27	64.3	0.03	Reference					Reference			
	Upper extremity function	Tertile 1 (<48)	35	34	97.1		1.7	1.3	2.3	<0.01		1.2	0.9	1.7	0.19
		Tertile 2 (48–74)	33	27	81.8		1.5	1.1	2.0	0.02		1.2	0.9	1.6	0.11
		Tertile 3 (>74)	39	22	56.4	<0.01	Reference					Reference			
	Lower extremity function	Tertile 1 (<14)	31	31	100		2.0	1.5	2.7	<0.01		1.6	1.1	2.4	<0.01
		Tertile 2 (14–55)	32	30	93.8		1.9	1.4	2.6	<0.01		1.5	1.1	2.0	<0.01
		Tertile 3 (>55)	44	22	50.0	<0.01	Reference					Reference			
	Bladder function	Tertile 1 (<51)	32	30	93.8		1.5	1.2	1.9	<0.01		1.2	0.9	1.5	0.13
		Tertile 2 (50–81)	34	27	79.4		1.3	0.9	1.7	0.13		1.1	0.9	1.5	0.30
		Tertile 3 (>81)	41	26	63.4	<0.01	Reference					Reference			
	Quality of life	Tertile 1 (<34)	34	32	94.1		1.8	1.3	2.5	<0.01		1.3	0.9	1.7	0.17
		Tertile 2 (34–50)	37	32	86.5		1.6	1.2	2.3	<0.01		1.4	1.0	1.8	0.05
		Tertile 3 (>50)	36	19	52.8	<0.01	Reference					Reference			

* All variables were included in the Table. ASA-PS, American society of anesthesiologists physical status; BMI, body mass index; JOACMEQ, JOA cervical myelopathy evaluation questionnaire; PR, prevalence ratio; 95%CI, 95% confidence interval.
